# Autologous T cells expressing the oncogenic transcription factor KLF6-SV1 prevent apoptosis of chronic lymphocytic leukemia cells

**DOI:** 10.1371/journal.pone.0192839

**Published:** 2018-02-12

**Authors:** Parviz Kokhaei, Mohammad Hojjat-Farsangi, Fariba Mozaffari, Ali Moshfegh, Fatemeh Pak, Ali Rashidy-Pour, Marzia Palma, Lotta Hansson, Anders Österborg, Håkan Mellstedt

**Affiliations:** 1 Immune and Gene Therapy Laboratory, Cancer Centre Karolinska, Department of Oncology and Pathology, Karolinska Institute, Stockholm, Sweden; 2 Cancer Research Center and Department of Immunology, Semnan University of Medical Sciences, Semnan, Iran; 3 The Persian Gulf Marine Biotechnology Medicine Research Center and Department of Immunology, School of Medicine, Bushehr University of Medical Sciences, Bushehr, Iran; 4 Physiology Research Center and Department of Physiology, Semnan University of Medical Sciences, Semnan, Iran; 5 Department of Hematology, Karolinska University Hospital Solna, Stockholm, Sweden; University of Manitoba, CANADA

## Abstract

Crosstalk between leukemic cells and the tumor microenvironment is of importance in chronic lymphocytic leukemia (CLL). T cells seem to sustain the survival of CLL cells by various mechanisms. The Krüppel-like family of transcription factors (KLFs) are identified as regulators of proliferation and cell death. In the present study, we analyzed the expression of the wild type (WT) gene KLF6 and the oncogenic splice variant 1 (KLF6–SV1) at the mRNA level in subsets of T cells from CLL patients (n = 29), multiple myeloma patients (n = 6) and normal donors (n = 10). RNA Silencing was used for wtKLF6 and KLF6-SV1. Tumor cell apoptosis was measured. A significant overexpression of wtKLF6 and KLF6-SV1 in T cells of CLL patients compared to normal donors and myeloma patients was noted (p<0.002). Western blot showed that both wtKLF6 and KLF6–SV1 were expressed in purified T cells from CLL patients. KLF6-SV1 siRNA transfection induced a significant down-regulation of KLF6-SV1 in CLL T cells, which lost the capability to sustain the growth of leukemic cells. However, no such a significant effect was seen after wtKLF6 transfection of the autologous T cells. The results suggest that KLF6-SV1 may play a role in the regulation of survival CLL cells.

## Introduction

Chronic lymphocytic leukemia (CLL) is characterized by the accumulation of CD5^+^, CD19^+^, CD23^+^ neoplastic small B cells in secondary lymphoid tissues and peripheral blood. In lymph nodes, the CLL clone proliferates in distinct areas called "proliferation centers" or "pseudo follicles" [1–4], with a low proliferative rate, accounting for approximately 1% to 2% of the tumor clone [5]. Persistence of the tumor clone is sustained by the ability to resist apoptosis rather than the proliferation rate (5). However, *in vitro* purified CLL cells undergo rapid spontaneous apoptosis, suggesting that *ex vivo* conditions may lack survival factors for leukemic cells might be present *in vivo* and that resistance to apoptosis is not only intrinsic to the leukemic B cells [6]. Dividing CLL cells were shown to express survivin and were frequently surrounded by T cells as well as other accessory stromal cells [7]. The tumor microenvironment seems to play an important role in pathology of CLL and may also be a target for treatment strategies [8]. Such treatment approaches may include inhibition of the crosstalk between CLL cells and the supportive microenvironment to overcome stromal-mediated tumor cell survival [9, 10].

We have recently completed a thorough analysis of shown an aberrant expression of cell surface and signaling molecules in T cells of CLL patients as well as an altered gene expression profile and increased production of cytokines as IL-4 and IFN-γ were also observed [11–13]. Functionally abnormal T cells may contribute to a microenvironment in which the proliferating leukemic clone resists differentiation and apoptosis sustaining the malignant phenotype of the B cells [12, 14]. In spite of the current knowledge of T cell support for CLL cells, the mechanisms of the anti-apoptotic effects of T cells are not fully understood [4, 12].

Kruppel-like factor (KLF) 6 belongs to the KLF family consisting of 17 proteins acting as DNA-binding transcription factors [15]. Wild-type KLF6 (wtKLF6) is a tumor-suppressor gene frequently inactivated in colorectal, prostate, colon cancers as well as in astrocytic gliomas [16, 17]. KLF6 interacts with cyclin D1 to mediate growth inhibition [18].

The KLF6 splice variant 1 (SV1) has 21 unique amino acids in the C-terminal, resulting in the loss of three zinc finger DNA binding domains [19]. The KLF6-SV1 N-terminal might activate the Ras/PI3-K/Akt proto-oncogenes [20]. Overexpression of c-Myc may act synergistically with KLF6-SV1 to increase the metastatic capacity of tumor cells [21]. KLF6-SV1 expression in tumor cells was associated with epithelial mesenchymal transition (EMT) and metastasis [17, 19]. It regulates extracellular matrix components as E-cadherins [22, 23]. The expression is associated with a poor prognosis of many types of cancers [3, 19, 24, 25]. It has also been shown that small interfering RNA (siRNA) down-regulating KLF6-SV1 reduced the capacity of tumor cells to progress *in vitro* and *in vivo* as well as enhanced the sensitivity to chemotherapy [26, 27]. KLF6-SV1 down-regulation in non-malignant cells might induce proliferation arrest, further indicating that KLF6-SV1 might have a role in cell growth [21].

In the present study we analyzed the expression of wtKLF6 and KLF6-SV1 in T cells of CLL patients and report a supportive effect of T cells expressing KLF6-SV1 on the survival of leukemic cells *in vitro*. No such effect was observed with T cells from patients with multiple myeloma (not considered to be dependent on T cells for tumor cell survival [28]) nor with T cells from healthy donors.

## Materials and methods

### Patients

Peripheral blood was collected from 29 patients with CLL, 6 patients with multiple myeloma and 10 healthy donors. The study was approved by The Regional Ethical Review Board in Stockholm: www.epn.se. The trial was performed in accordance with the Helsinki declaration on the use of human samples for clinical research. Sampling was done after written and oral informed consent was obtained from the participants. Diagnostic criteria for CLL and multiple myeloma have been described previously [29]. The characteristics of the patients are shown in Table 1.

**Table 1:** Characteristics of CLL and multiple myeloma patients.

Patient’s number	Age	Sex	Clinical stage*	M-component type & concentration (g/L)	Previous therapy	Disease phase	CD3%	Time since last therapy (mo)
**CLL patients**								
**CLL1**	71	M	I	NA	Non	Indolent	19	NA
**CLL2**	73	F	I	NA	Non	Indolent	5.5	NA
**CLL3**	72	M	0	NA	Non	Indolent	34	NA
**CLL4**	58	M	I	NA	Non	Indolent	3.5	NA
**CLL5**	63	M	0	NA	Non	Indolent	17.6	NA
**CLL6**	84	M	I	NA	Non	Indolent	7	NA
**CLL7**	59	M	0	NA	Non	Indolent	15	NA
**CLL8**	74	F	II	NA	Non	Indolent	1.8	NA
**CLL9**	68	F	II	NA	Non	Indolent	4.4	NA
**CLL10**	78	F	II	NA	Non	Indolent	12	NA
**CLL11**	63	M	0	NA	Non	Indolent	1.5	NA
**CLL12**	76	M	I	NA	Non	Indolent	19	NA
**CLL13**	68	M	I	NA	CLB	Response/Plateau	0.4	12
**CLL14**	71	F	0	NA	None	Indolent	24	NA
**CLL15**	65	F	I	NA	F	Response/Plateau	3.4	1
**CLL16**	72	M	0	NA	None	Indolent	21	NA
**CLL17**	73	M	I	NA	None	Indolent	15.7	NA
**CLL18**	86	F	I	NA	None	Indolent	13	NA
**CLL19**	79	M	I	NA	None	Indolent	13.4	NA
**CLL20**	69	F	I	NA	None	Indolent	1.8	NA
**CLL21**	65	M	II	NA	CLB	Response/Plateau	5.5	24
**CLL22**	79	F	I	NA	CLB	Response/Plateau	ND	4
**CLL23**	73	F	II	NA	None	Indolent	9.7	NA
**CLL24**	75	M	II	NA	None	Indolent	67.5	NA
**CLL26**	66	F	II	NA	None	Indolent	2	NA
**CLL27**	71	F	0	NA	None	Indolent	6.8	NA
**CLL28**	69	M	0	NA	None	Indolent	9.1	NA
**CLL29**	78	F	0	NA	None	Indolent	ND	NA
**Myeloma patients** **Group**								
**MM1**	73	F	IA	IgA, 20	None	Asymptomatic	ND	NA
**MM2**	77	M	IA	IgG, 7	None	Asymptomatic	ND	NA
**MM3**	75	F	IIA	IgG, 23	MP	Response/Plateau	ND	49
**MM4**	83	F	IIIA	IgG, 70	MP	Response/Plateau	ND	9
**MM5**	85	F	IIIA	IgG, 8	MP	Response/Plateau	ND	51
**MM6**	79	F	IIA	IgA, 23	None	Asymptomatic	ND	NA

* Rai (CLL) and ISS (MM) staging system were used

NA = not applicable, CLB = Chlorambucil, F = Fludarabine, MP = Melphalan-Prednisone, ND = not done

### Purification of T and B cells

Peripheral blood mononuclear cells (PBMC) were isolated from heparinized blood as described [30]. CLL B cells from PBMC were depleted by filtration through a nylon wool column (Biotest, Breiech, Germany) [31] and T cells were further enriched by immunomagnetic depletion of B cells, NK cells and monocytes using MidiMACS columns and anti-CD19, anti-CD56 and anti-CD14 MACS MicroBeads (MiltenyiBiotec, Bergisch Gladbach, Germany) according to the manufacturer's recommendations. The purity of CD3 T-cells was 93–99% as determined by flow cytometry. CD4^+^ and CD8^+^ T cell were further purified from CD3^+^ T cells using the MACS MicroBeads negative selection kit (MiltenyiBiotec, Bergisch Gladbach, Germany). The purity of CD4^+^ and CD8^+^ cells were 96% and 94%, respectively. The purity of B cells (CD19^+^) was >99% as determined by flow cytometry.

### Cellular staining and flow cytometry

Surface markers were analysed by flow-cytometry using fluorochrome-conjugated monoclonal antibodies. Anti-CD3, CD4, CD8, CD19, and CD56 monoclonal antibodies were purchased from BD Biosciences (San Jose, CA, USA). Appropriate concentrations of antibodies as well as isotype -matched controls were added to the cells (5 x 10^5^ cells/ tube) in 100 μL staining buffer and incubated for 25 min at 4°C in the dark. Analyses were performed using a FACS Canto II flow cytometry (BD) and the FLOW JO™ software (Tree Star, Ashland, OR, USA). A minimum of 10,000 lymphocyte-gated cells, were acquired and analyzed for CD3^+^CD4^+^, CD3^+^CD8^+^, CD19^+^ and CD56^+^ cells. Criteria for positive staining was set at a fluorescent intensity displayed by <0.5% of the cells stained by the appropriate fluorochrome-conjugated isotype control mAb.

### siRNA transfection

Purified T cells from CLL patients (1x10^5^) were cultured in 96-well cell culture plates in 100 μl Accell siRNA Delivery Media (Thermo scientific, PA, USA). Sequence specific siRNA for wtKLF6 (5’-GGGGAGGCAUCGCCAUUU-3’), KLF6-SV1 (5’-CAGGGAAGGAGAAAAGCCUUU-3’) [27, 32] and control siRNA (5’-UGGUUUACAUGUCGACUAA-3’) (Thermo scientific Dharmacon, PA, USA) were added to the cells (1 μM siRNA in Accell siRNA delivery medium, (Thermo Fisher Scientific) and incubated for 48 hours. Cells were then harvested. Total RNA was extracted and cDNA synthesized [33]. Apoptosis was measured after 72 hours by Annexin V/PI staining using flow cytometry (see below).

### Apoptosis assay

Untransfected purified CLL cells or T cells (1x10^5^) were cultured alone or after transfection with wtKLF6 siRNA, KLF6-SV1 siRNA as well as mock transfected for 72 hours. The cells were harvested and apoptosis measured by Annexin V/PI staining using a commercial kit (BD San Jose, CA, USA) according to the manufacturer's instructions [30]. Briefly, cells were washed in PBS and stained for surface CD19 and CD3 expression. After washing in PBS, cells were resuspended in binding buffer. Annexin V/PI (BD) was added and incubated for 15 min at room temperature in the dark. Cells were analyzed using BD FACS Canto II flow cytometer (BD) and the FLOW JO™ software (Tree stars). A minimum of 10000 gated events were analyzed.

### Isolation of RNA and cDNA synthesis

Total RNA was extracted from freshly isolated CD4^+^ and CD8^+^ T cells and CLL cells from CLL patients as well as from CD4^+^ and CD8^+^ T cells of multiple myeloma patients and healthy donors using the RNeasy mini kit (Qiagen, Hilden, Germany) according to the manufacturer’s instructions. The KLF6 negative cell line 293T (isolated from human embryonic kidneys, ATCC 30–2002) was cultured in Dulbecco’s Modified Eagle’s Medium (DMEM) with 10% FBS (heat inactivated), 2mM L-glutamine 1% and penicillin/streptomycin. The cells were washed and RNA extracted. The purity and quality of extracted RNA were confirmed by measuring the A260/A280 ratio and separation on agarose gel to ensure RNA integrity prior to cDNA synthesis. cDNA synthesis was performed by converting total RNA to cDNA, using a cDNA kit (Fermentas/Thermo) (Waltham, Massachusetts, USA) according to manufacturer's guidelines. Briefly, 100 ng of total RNA and 50 pmol/μl of random hexamer primers and 50 pmol/μl of oligo dT primers were heated in 11 μl of RNase-free water at 65C for 10 min and chilled in ice water. A mixture consisting of 4 μl of 5x RT buffer, 2 μl of 20 mM DTT, 1 μl of 10mM dNTPs, and 1 μl of RNase Inhibitor (RiboLock RNase Inhibitor (20U/μL); Waltham, Massachusetts, USA) were added and incubated at room temperature for 5 min. One microliter of MMLV Reverse Transcriptase (200 U/μl; thermo Scientific) was added to the reaction and incubated at 42°C for 60 min.

### Quantitative real-time RT-PCR (qRT-PCR)

The expression of the KLF6-SV1 gene was quantified by qRT-PCR. Total RNA was extracted from purified CD4^+^ and CD8^+^ T-cells of CLL patients, multiple myeloma patients and healthy donors. qRT-PCR was performed using the TaqMan probe (Life technology, Stockholm, Sweden) and the 79000 Real Time PCR equipment (ABI, CA, USA). qRT-PCR reactions were performed in 20 μL in duplicate. Analysis of sequences of interest was performed by the comparative Ct method of relative quantification using GAPDH as endogenous control and PBMC of a normal donor as calibrator. The difference in Cq values (mean of duplicates) between the investigated transcript and the endogenous reference gene was calculated, and the mean normalized expression (MNE) reported as 2^-ΔCq^.

### Immunoblotting

Purified T cells from CLL patients and healthy donors (5 x 10^6^) were lysed in buffer containing 1% Triton X-100, 50 mMTris-HCl pH 7.4, 150 mMNaCl, 5 mM EDTA, and 1% protease inhibitor cocktail. Gel electrophoresis was run applying 20–30 μg of cell lysate per lane using a pre-cast NuPAGE® 10% Bis-Tris Gel (Invitrogen, Carlsbad, CA, USA) at 200 V for 1 hour under reducing conditions. Resolved proteins were transferred onto polyvinylidene difluoride (PVDF) microporous membranes (Millipore, Billerica, MA, USA) in a Mini Trans-Blot cell (Thermo Fisher Scientific). Membranes were blocked overnight with 5% nonfat milk (Semper, Stockholm, Sweden) in PBS with 0.05% Tween 20 (PBS-T). All immunostaining steps and washings were performed in PBS-T supplemented with 5% nonfat milk (Semper). Membranes were incubated with the primary antibody overnight. The following mAbs were used for detection of proteins: anti-KLF6 and anti-KLF6-SV1 (Santa Cruz Biotechnology Inc., Santa Cruz, CA, USA). After extensive washings, membranes were incubated with the appropriate HRP-conjugated antibody (DAKO, Glostrup, Denmark) for 1 hour. Membranes were then developed using enhanced chemiluminescence Amersham ECL Select Detection Reagents (Amersham Place, Little Chalfont, UK) according to manufacturer’s instruction. Membranes were subsequently stripped and re-probed using an anti-β-actin antibody as loading control.

### Statistical analysis

Student's *t-*test was used for comparison between the groups. The influence of variables on apoptosis was assessed by the non-parametric Mann Whitney test. The non-parametric Wilcoxon signed rank test was used for dependent groups. A p-value <0.05 was considered significant.

## Results

### Wild-type KLF6 and KLF6-SV1 gene expression in CLL

**Fig 1:**
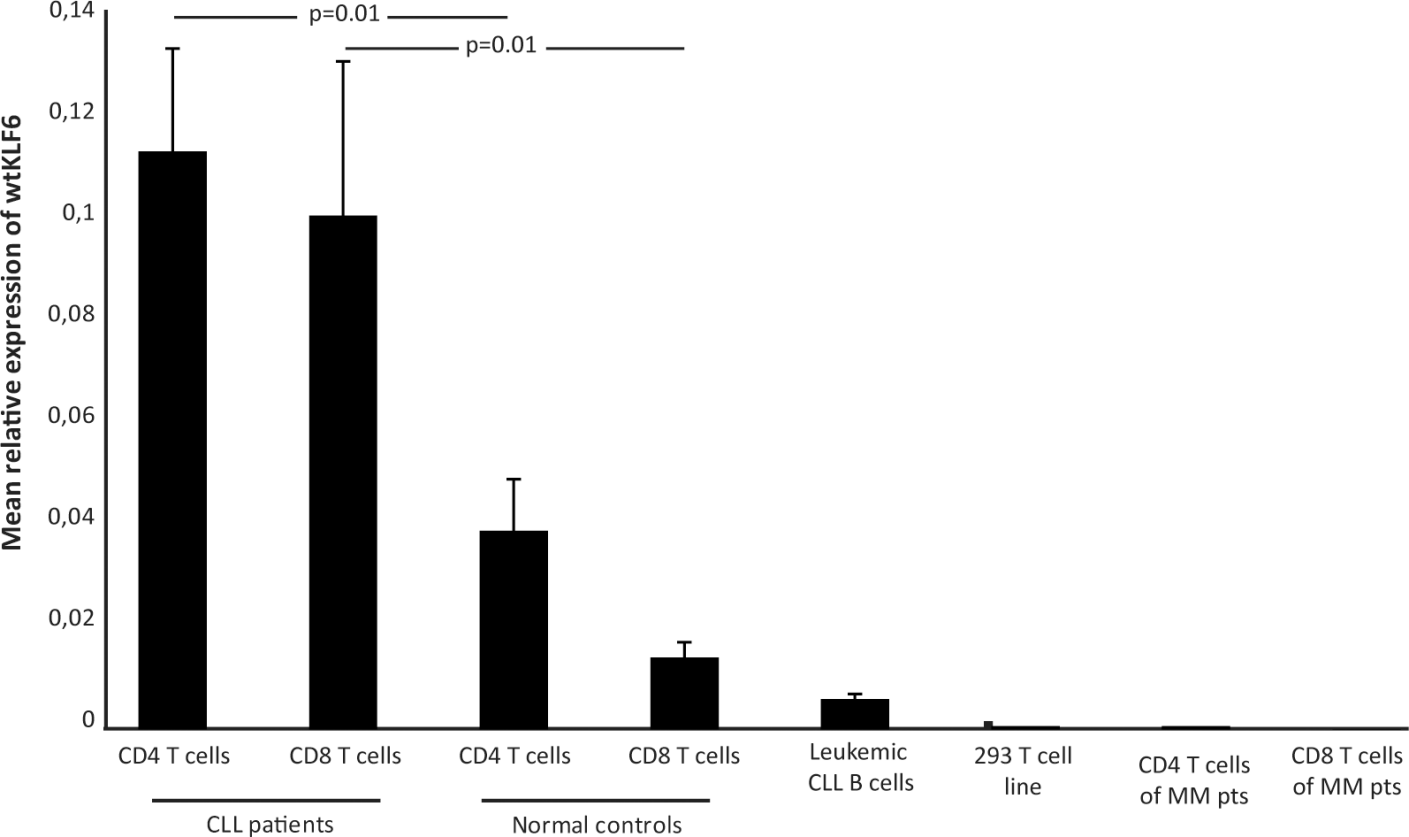
wtKLF6 mRNA expression in purified CD4+ and CD8+ T cells of CLL patients (n = 29), B cells of CLL patients (n = 6), CD4+ and CD8+ T cells of myeloma patients (n = 6) and healthy donors (n = 10) as well as in the 293 T cell line.

**Fig 2:**
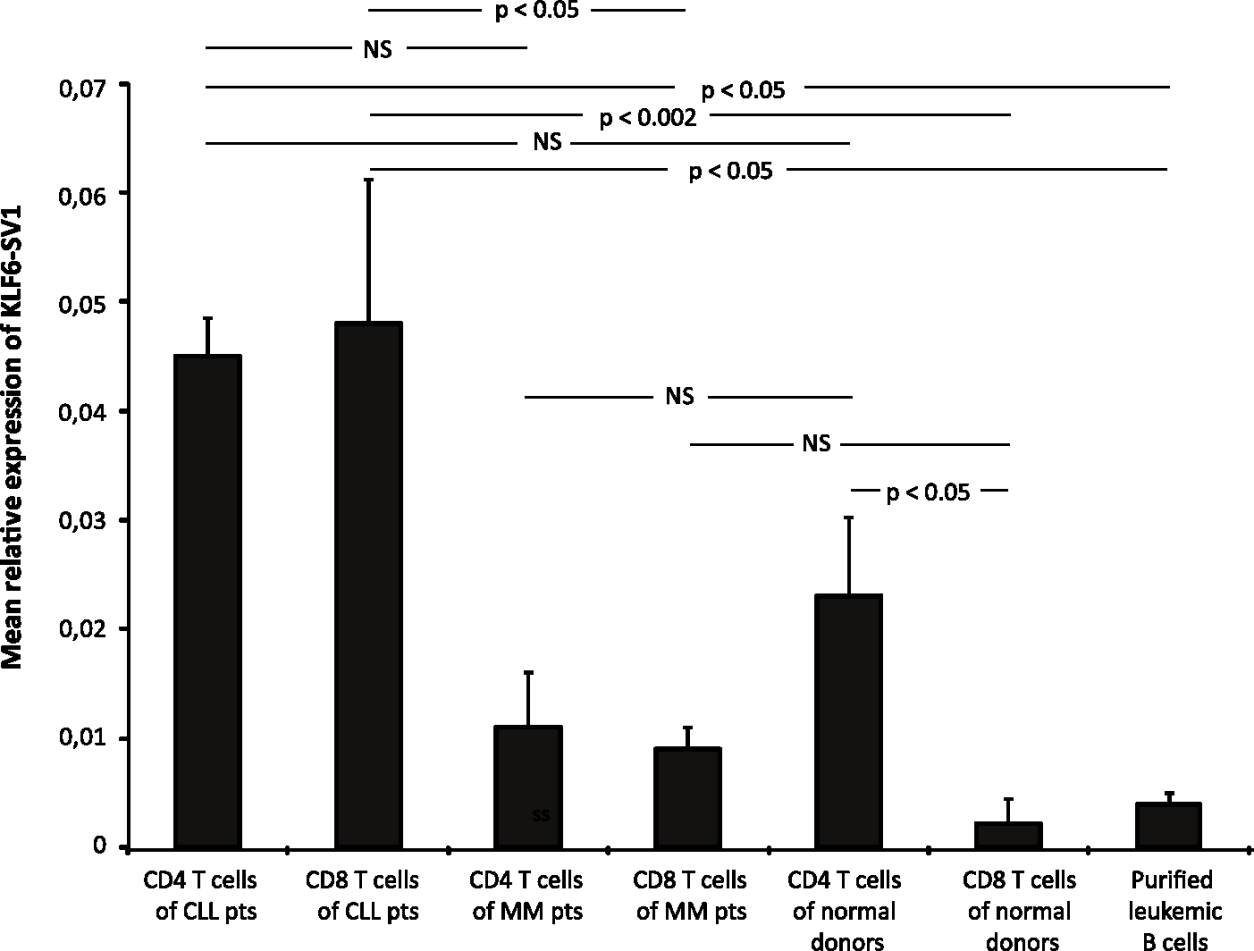
KLF6-SV1 mRNA expression in purified CD4^+^ and CD8^+^ T cells of CLL patients (n = 29), myeloma patients (n = 6) and healthy donors (n = 10) as well as in purified leukemic B cells of CLL patients (n = 6).

The relative gene expression of wtKLF6 was statistically significantly higher in CD4^+^ and CD8^+^ T-cells of CLL patients as compared to healthy donors (p = 0.01) (Fig 1). There was no difference between CD4^+^ and CD8^+^ T cells. Expression of wtKLF6 in CLL B-cells was low as well as in the T-cell line 239 (negative control) (www.abnova.com). The relative gene expression of KLF6-SV1 was statistically significantly higher in CD8^+^ T-cells of CLL patients as compared to CD8^+^ T cells derived of myeloma patients (p<0.05) and healthy donors (p = 0.002) (Fig 2). There was a trend towards a lower expression of KLF6-SV1 in CD4 T cells of myeloma patients as compared to CD4 T cells of CLL patients (p = 0.09) but not compared to healthy donors. There was no significant difference in KLF6-SV1 gene expression comparing CD4^+^ and CD8^+^ T cells of CLL patients. Furthermore, CD4^+^ T cells of healthy donors expressed a higher level of KLF6-SV1 as compared to CD8^+^ T cells (p <0.05). Significantly lower level of KLF6-SV1 was observed (Student's t-test) in leukemic CLL cells as compared to both CD4^+^ and CD8^+^ T cells of CLL patients (p< 0.05) (Fig 2).

### KLF6-SV1 protein expression

**Fig 3:**
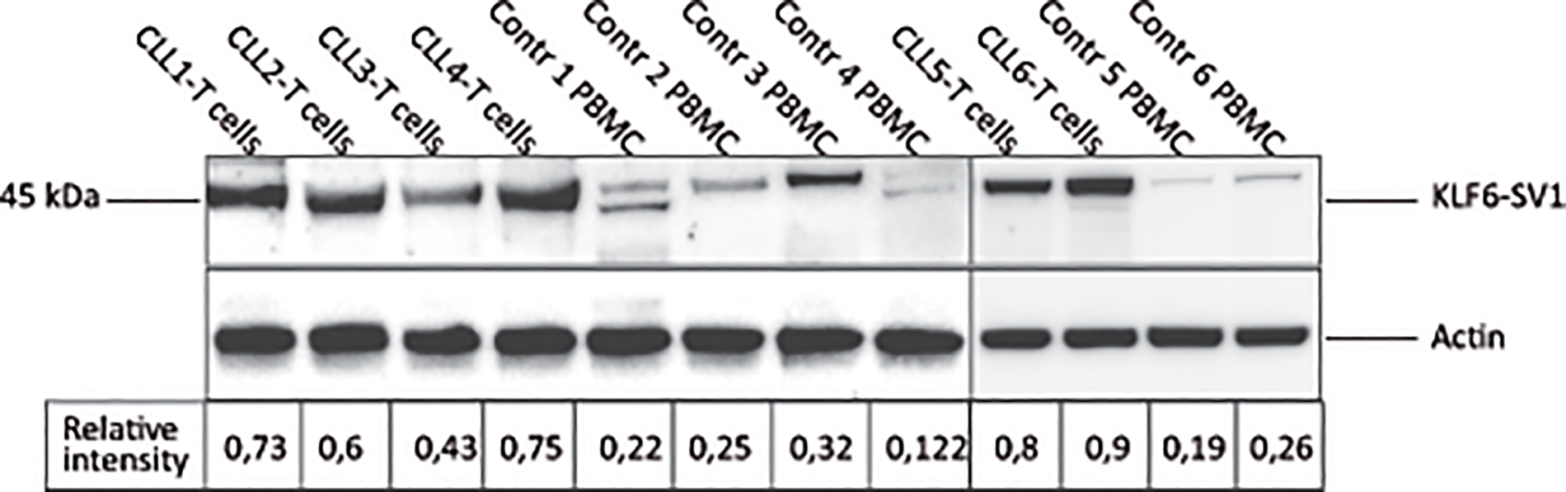
KLF6-SV1 protein expression (Western blot) in purified CLL T (CD3^+^) cells of CLL patients (n = 6) as well as PBMC (n = 4) and purified T cells (n = 2) of healthy donors. Relative intensity was measured in relation to β-actin.

The relative expression of the KLF6-SV1 protein was analyzed in purified CLL T cells (CD3^+^) (n = 6), compared to PBMC (n = 4) and purified T cells from healthy donors (n = 2). There was a significantly higher protein expression in CLL T cells as compared to PBMC and T cells of healthy donors (p = 0.01, Mann Whitney test) (Fig 3).

### wtKLF6 and KLF6-SV1 siRNA transfection of CLL T cells and impact on CLL B-cell apoptosis

**Fig 4:**
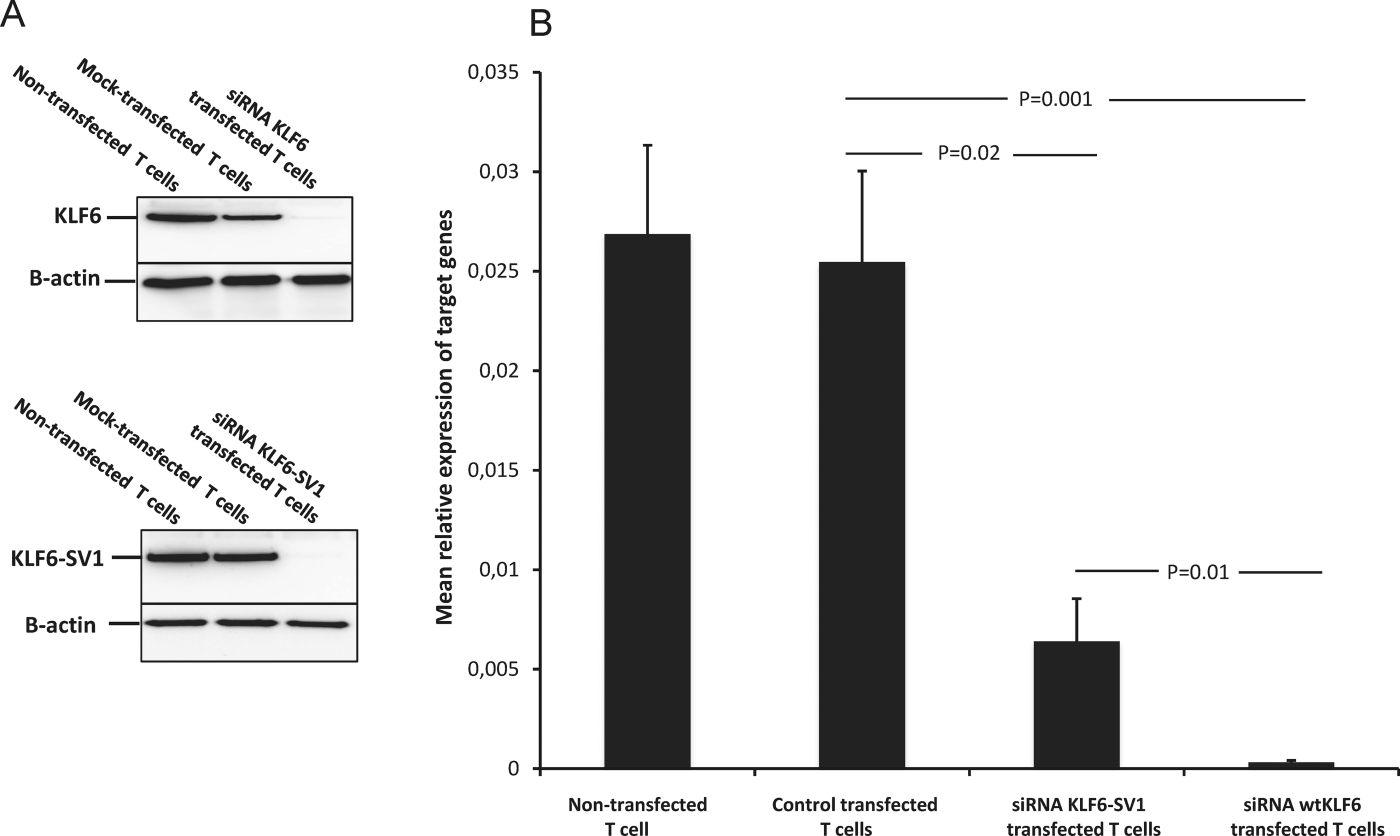
A) Downregulation of KLF6 and KLF6-SV1 protein expression in T cells of CLL patients (representative experiment). B) KLF6-SV1 and wtKLF6 siRNA transfection of T cells from CLL patients (n = 4) induced a dose-dependent decrease of specific mRNA after 48h of incubation. An optimal dose of siRNA (1μM) was used.

T cells from CLL patients were transfected with KLF6-SV1 and wtKLF6 siRNA with a high efficiency (Fig 4A). Transfection with KLF6-SV1 siRNA and wtKLF6 siRNA respectively induced a significant decrease in the gene and protein expression of KLF6-SV1 and wtKLF6 as exemplified in Fig 4A and 4B. No significant decrease in target mRNA and protein expression of non-transfected or control-transfected cells was noted.

**Fig 5:**
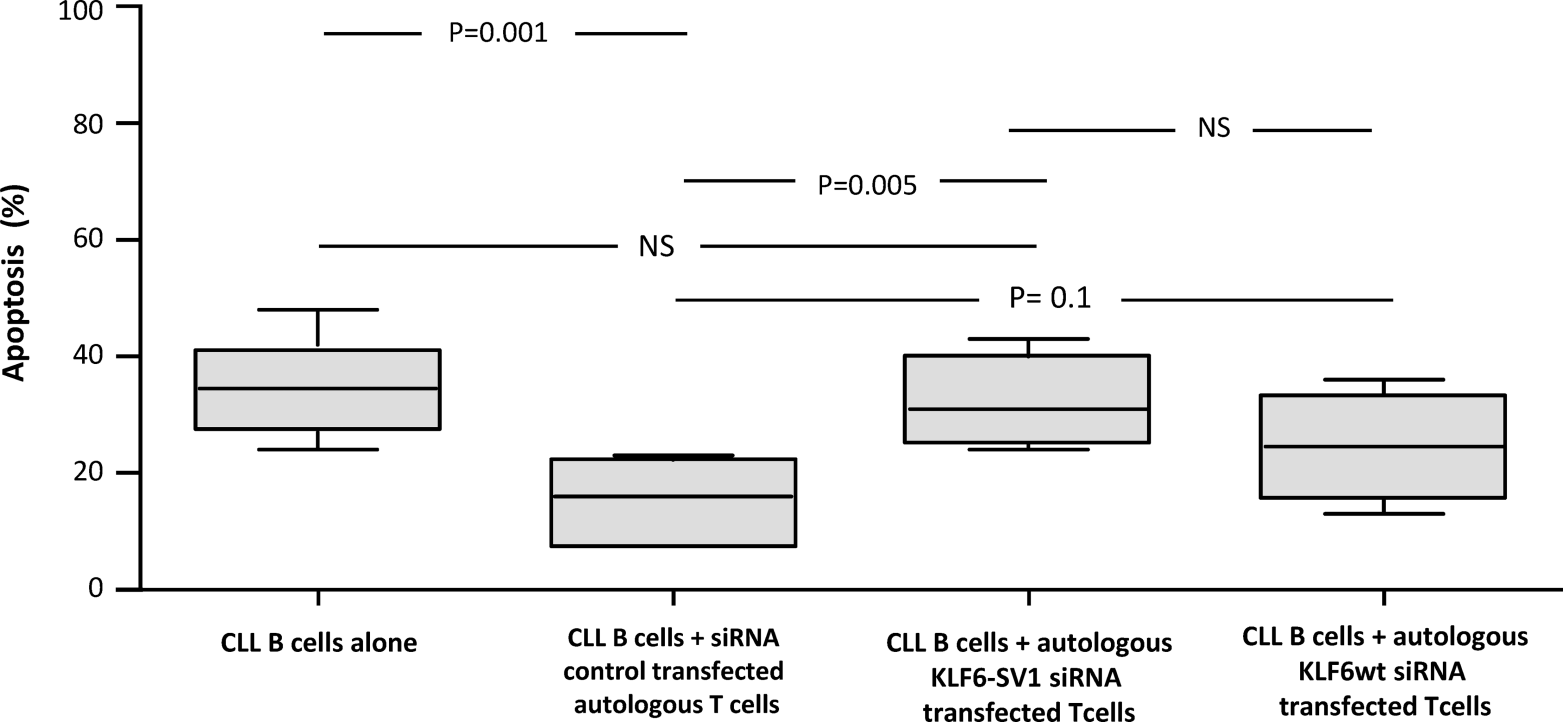
Apoptosis of CLL cells (CD19^+^ cells) co-cultured with siRNA control transfected, transfected KLF6-SV1 and wtKLF6 autologous T cells (n = 6) for 48 hours. The box represents the 25th to 75th percentiles with line at median. The top whisker is drawn from the value associated with the 75th to 90th percentile, and bottom from 25th to 10th percentile. P-values are indicated at the top.

After culture of purified leukemic CLL cells alone for 48 h, 38% (median) apoptotic cells were noted (Fig 5). When the leukemic cells were co-cultured with non-siRNA-transfected autologous T cells (ratio 1:1) a significant protection from apoptosis of the leukemic CLL cells was noted. Only 16% (median) apoptotic cells was seen (p = 0.001) (Fig 5). However, when the leukemic B-cells were co-cultured with autologous T cells transfected with KLF6-SV1 siRNA the protective effect of T-cells was lost. The percentage of apoptotic leukemic cells noted after 48 h of culture was comparable to that of leukemic cells cultured alone. The difference in surviving B cells comparing B cells co-cultured with control-transfected T cells and those transfected with KLF6-SV1 siRNA was statistically significant (p = 0.005) (Fig 5). When T cells were transfected with wtKLF6 siRNA, no significant loss of the protective effect on the survival of the CLL cells was noted (p = 0.1, Mann Whitney test).

## Discussion

CLL cells frequently undergo rapid and spontaneous apoptosis *in vitro* under conditions that support the growth of normal human B-cells. This may suggest that extrinsic factors *in vivo* might be of importance for CLL cell survival and that resistance to apoptosis is not only intrinsic of the CLL cells. Our previous studies have demonstrated that autologous T cells of CLL patients supported survival of leukemic cells *in vitro* (12). CLL T cells produced high levels of IL-4 and IL-5, which were of importance for the survival of CLL cells [12, 29–31]. We could also show that the supportive effect of the T cells was mediated through soluble factors as well as by a direct cell-cell contact in a T cell dose-dependent manner. High doses of IL-4 inhibited apoptosis of CLL cells *in vitro*, but to a lower degree compared to that of autologous T cells [12]. The anti-apoptotic effects of the micro-environment were associated with upregulation of anti-apoptotic factors at BCL-XL and MCL-1 mediated by activated autologous T cells and macrophages [34].

Studies in several malignancies have shown that KLF6-SV1 regulated the tumor cell cycle and survival [19, 22, 35]. KLF6-SV1 antagonized both the tumor-suppressor wtKLF6, and the pro-apoptotic protein NOXA, by targeting those proteins inhibiting a rapid enzymatic degradation [9]. The NOXA/MCL-1 balance in CLL cells was inverted in lymph nodes compared to peripheral blood, indicative of an increased resistance of CLL cells in lymph nodes as compared to peripheral blood[34]. KLF6-SV1 may also promote EMT, contributing to the acquisition of a highly metastatic tumor phenotype. High expression of KLF6-SV1 in primary breast tumors was also a prognostic factor for increased risk of metastasis and a poor survival [9, 36].

T cell-derived cytokines, including IL-1β, TNF-α and IFN-γ, enhanced significantly TGF-β1-induced EMT in breast cancer (FMC-7) and lung cancer (A549) cell lines (33). TGF-β has also been shown to be produced both by the leukemic B cells and the T cells in CLL, as well as being detected in serum of CLL patients [37]. TGF-β may normally process growth inhibitory effects but is dysregulated in malignancies. CLL cells have been shown to be resistant to apoptotic effects of TGF-β [38]. Other mechanisms than TGFβ might also be considered to be involved in the T cell promoting survival effects on CLL cells. We have previously shown that the wtKLF6 gene was differentially expressed in CLL cells as compared to normal T cells (12).

In the present study, we could confirm that CD4^+^ and CD8^+^ T cells in CLL expressed high levels of wtKLF6 compared to healthy donors. Moreover, an increased expression of KLF6-SV1 mRNA and protein in at least CD4^+^ T cells from CLL patients compared to T cells from myeloma patients and normal healthy donors was noted. Silencing of the KLF6-SV1 gene in CLL cells significantly abrogated the T cell mediated inhibition of apoptosis of CLL cells. It might be assumed that KLF6-SV1 may play a role in the survival supportive effects of autologous T cells for CLL cells. This effect seemed to be specific for CLL T cells as T cells from myeloma patients; another B malignancy did not express KLF6-SV1. Moreover, T cells in myeloma might not significantly contribute to the anti-apoptotic effects of myeloma plasma cells (38).

KLF6-SV1 down regulation in the ovarian carcinoma cell line SKOV-3 inhibited tumor growth *in vivo* [24] and silencing of KLF6-SV1 induced apoptosis *in vivo* and *in vitro*, as well as restored chemosensitivity of prostatic carcinoma cells [39]. KLF6-SV1 siRNA transfection also induced suppression of gastric cancer cell growth, colony formation, migration and invasion [27]. Apoptosis was also noted in KLF6-SV1 siRNA transfected lung cancer cells [26]. Our observation that KLF-6 SV1 expression was highly significantly lower in CLL leukemic cells compared to T cells might be interesting as overexpression of KLF6-SV1 in human cancer cells may accelerate cancer progression and metastasis in animal models as well as in human cancer [39], [19].

Our data is the first report suggesting that KLF6-SV1 may be involved in an anti-apoptotic effect exerted by T cells on CLL B cells. T cells of multiple myeloma patients, another chronic B lymphocyte malignancy, did not express high level of this oncogenic proteins further supporting the notion that KLF6-SV1 in T cells from CLL might be part of the pathobiology of CLL as also suggested by others [40]. A number of studies have highlighted the potential efficacy and specificity of future siRNA/RNAi-based therapies in malignancies through up-regulation of the pro-apoptotic protein Noxa and reduction of the anti-apoptoptic proteins MCL-1 and Bim [3, 24]. Targeting T cells might be a strategy to increase tumor cell apoptosis in CLL. Further studies are warranted to assess the role of T cell mediated regulation of the CLL clone.
